# Single‐molecule epiallelic profiling of DNA derived from routinely collected Pap specimens for noninvasive detection of ovarian cancer

**DOI:** 10.1002/ctm2.1778

**Published:** 2024-07-31

**Authors:** Christine M. O'Keefe, Yang Zhao, Leslie M. Cope, Chih‐Ming Ho, Amanda N. Fader, Rebecca Stone, James S. Ferris, Anna Beavis, Kimberly Levinson, Stephanie Wethington, Tian‐Li Wang, Thomas R. Pisanic, Ie‐Ming Shih, Tza‐Huei Wang

**Affiliations:** ^1^ Department of Biomedical Engineering Johns Hopkins University Baltimore Maryland USA; ^2^ Sidney Kimmel Comprehensive Cancer Center Johns Hopkins University School of Medicine Baltimore Maryland USA; ^3^ Departments of Oncology and Biostatistics Johns Hopkins University School of Medicine Baltimore Maryland USA; ^4^ Gynecologic Cancer Center Department of Obstetrics and Gynecology Cathay General Hospital Taipei Taiwan; ^5^ School of Medicine Fu Jen Catholic University New Taipei Taiwan; ^6^ Department of Gynecology and Obstetrics Johns Hopkins University School of Medicine Baltimore Maryland USA; ^7^ Greater Baltimore Medical Center Towson Maryland USA; ^8^ Department of Pathology Johns Hopkins University School of Medicine Baltimore Maryland USA; ^9^ Institute for NanoBioTechnology Johns Hopkins University Baltimore Maryland USA; ^10^ Department of Mechanical Engineering Johns Hopkins University Baltimore Maryland USA

**Keywords:** digital melt, DNA methylation, microfluidics, ovarian cancer, Pap smear

## Abstract

Recent advances in molecular analyses of ovarian cancer have revealed a wealth of promising tumour‐specific biomarkers, including protein, DNA mutations and methylation; however, reliably detecting such alterations at satisfactorily high sensitivity and specificity through low‐cost methods remains challenging, especially in early‐stage diseases. Here we present PapDREAM, a new approach that enables detection of rare, ovarian‐cancer‐specific aberrations of DNA methylation from routinely‐collected cervical Pap specimens. The PapDREAM approach employs a microfluidic platform that performs highly parallelized digital high‐resolution melt to analyze locus‐specific DNA methylation patterns on a molecule‐by‐molecule basis at or near single CpG‐site resolution at a fraction (< 1/10th) of the cost of next‐generation sequencing techniques. We demonstrate the feasibility of the platform by assessing intermolecular heterogeneity of DNA methylation in a panel of methylation biomarker loci using DNA derived from Pap specimens obtained from a cohort of 43 women, including 18 cases with ovarian cancer and 25 cancer‐free controls. PapDREAM leverages systematic multidimensional bioinformatic analyses of locus‐specific methylation heterogeneity to improve upon Pap‐specimen‐based detection of ovarian cancer, demonstrating a clinical sensitivity of 50% at 99% specificity in detecting ovarian cancer cases with an area under the receiver operator curve of 0.90. We then establish a logistic regression model that could be used to identify high‐risk patients for subsequent clinical follow‐up and monitoring. The results of this study support the utility of PapDREAM as a simple, low‐cost screening method with the potential to integrate with existing clinical workflows for early detection of ovarian cancer.

**Key points:**

- We present a microfluidic platform for detection and analysis of rare, heterogeneously methylated DNA within Pap specimens towards detection of ovarian cancer.
- The platform achieves high sensitivity (fractions <0.00005%) at a suitably low cost (∼$25) for routine screening applications.
- Furthermore, it provides molecule‐by‐molecule quantitative analysis to facilitate further study on the effect of heterogeneous methylation on cancer development.

## Graphical abstract

- Ovarian cancer cells exhibit heterogeneous methylation patterns that could be leveraged as biomarkers for early detection.
- Cellular fragments and debris from ovarian tumors migrate to the cervix, where they are accessible via Pap specimen collection.
- The microfluidic HYPER‐melt platform digitizes bisulfite‐converted DNA into thousands of nanochambers to isolate heterogeneous epialleles, and uses digital high resolution melt to profile methylation patterns.
- A bioinformatic pipeline combines patterns from a multi‐gene panel into a methylation density score that gives the probability that a given sample contains ovarian cancer DNA.

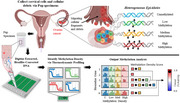

## 1 INTRODUCTION

Pelvic high‐grade serous carcinoma (HGSC) remains the most aggressive gynecologic cancer and the fifth most common cause of cancer‐related death for women in the United States. Over 22,000 women in the United States are diagnosed with ovarian cancer annually and approximately 14,200 (> 60%) women die each year from this disease.^1^, ^2^ The poor survival of HGSC patients is largely attributable to delayed diagnosis, as approximately 75% of patients do not present symptoms until an advanced stage when curative resection is no longer possible. Indeed, various epidemiological studies have found that women whose ovarian cancer is diagnosed at an early stage have significantly higher 5‐year survival rates (> 90%) as compared to those diagnosed at late stages (∼ 30%).^3^

The significant improvement in survival among ovarian cancer patients diagnosed at the early stages of the disease has engendered considerable effort over the past several decades to develop tests for screening and early detection of HGSC. Most notably, a number of large‐scale screening trials based on protein biomarkers, such as cancer antigen‐125 (CA‐125), and/or imaging approaches, such as transvaginal ultrasound, have been conducted; however, these approaches have yet to demonstrate a meaningful survival benefit.^4^ Consequently, several national organizations, including the US Preventative Services Task Force, do not currently recommend routine screening for ovarian cancer due to the observation that “*the potential harms outweigh the potential* *benefits*”.^5^ There thus remains an urgent need for the development of novel, relatively non‐invasive diagnostic approaches that are not only able to reliably detect HGSC but to do so at sufficiently early stages to improve patient outcomes.^6^, ^7^, ^8^, ^9^, ^10^

The new paradigm in the genesis of HGSC posits that the majority of HGSCs likely originate in the fallopian tube^11^, ^12^, ^13^, ^14^, ^15^ from precursor lesions called serous tubal intraepithelial carcinoma (STIC). STIC cells are thought to be shed or migrate to the ovaries, where they rapidly evolve to form “ovarian” tumours and disseminate to the surrounding peritoneum and omentum to establish advanced malignancies. Concurrently, these atypical cells are thought to also travel in the opposite direction, from the fallopian tubes through the uterine cavity and down to the cervical canal.^16^ Thus, proximity fluid samples collected from cervical‐vaginal fluid^6^, ^7^, ^17^ in Pap specimens have been investigated for utility in the detection of ovarian cancer. Liquid‐based Pap sampling is relatively non‐invasive and has become a routine gynecologic procedure for detecting human papillomavirus in the screening of cervical cancer.

In addition to genetic abnormalities, studies by us and others have demonstrated that virtually all ovarian cancers exhibit aberrant DNA methylation when compared with histologically unremarkable tissues.^18^, ^19^, ^20^, ^21^ Biomarkers based on cancer‐specific DNA methylation hold promise for the early detection of cancer, as many cancer‐specific methylation alterations emerge during precancerous stages.^22^, ^23^ Indeed, we have recently reported a select set of loci whose hypermethylation is able to distinguish STIC and cancer tissues from histologically unremarkable gynecological epithelia with high accuracy.^21^ Yet despite these advantages, the detection of ultra‐rare DNA methylation biomarkers in complex samples such as Pap specimens remains technically challenging. In particular, while aberrant methylation is present in the vast majority of STIC lesions and HGSC tumours, the methylation patterns themselves typically arise progressively and in a stochastic manner, leading to clonal cell populations with heterogeneous methylation patterns and profiles. This poses considerable challenges for commonly employed polymerase chain reaction (PCR)‐based techniques, such as methylation‐specific PCR (MSP) and digital‐MSP (dMSP) that are designed to only detect *specific*, predefined methylation patterns (typically densely‐methylated). This drawback undermines the ability of these tests to detect and quantify heterogeneously‐methylated tumour DNA, particularly at early stages of the disease and in challenging samples such as liquid biopsies and Pap specimens. On the other hand, while targeted bisulfite next‐generation sequencing (NGS) is nominally able to assess heterogeneous methylation, it has yet to demonstrate sufficient sensitivity to reliably detect epiallelic fractions below 0.1% and remains relatively expensive for routine screening applications, requiring substantial expertise, sample prep costs and time.

To address this challenge, we previously developed an alternative approach called DREAMing that provides a facile and relatively inexpensive means of detecting and assessing ultra‐rare epiallelic variants such as those found in liquid biopsies.^24^ More recently, the DREAMing assay has been adapted for use with a microfluidic platform called HYPER‐melt,^25^ which demonstrated the ability to quantify heterogeneously‐methylated biomarkers at single‐copy sensitivity in a background of 2 million unmethylated templates. Parallelized digital high‐resolution melt is then performed to analyze molecule‐by‐molecule methylation heterogeneity within a sample. This simple‐to‐use platform achieves the high sensitivity, low cost and practicality necessary for routine clinical use.

In this study, we introduce a new platform called PapDREAM for the detection and analysis of rare, ovarian‐cancer‐specific aberrations of DNA methylation from routinely collected Pap specimens. PapDREAM employs a data analysis pipeline that leverages quantitative, multidimensional datasets provided by the HYPER‐Melt platform, derived from interrogation of the CpG methylation status on a molecule‐by‐molecule basis for each locus, to determine an overall predictive score for ovarian cancer. The pipeline readily scales from individual biomarkers to panels and provides a snapshot of tumour epigenetic heterogeneity that can be informative to further studies. Finally, we evaluated the methylation patterns of ovarian cancer‐specific methylation biomarkers in DNA derived from 25 healthy and 18 HGSC Pap specimens to assess whether such an approach could lead to improved clinical performance for routine screening applications.

## 2 RESULTS

### 2.1 Platform overview

The PapDREAM platform utilizes microfluidic thermodynamic profiling technology to identify ultra‐rare aberrant methylation patterns within Pap specimens. Previous studies examining the dynamics of DNA methylation have revealed that aberrant DNA methylation associated with cancer generally arises through the stochastic accrual (or loss) of methylation at particular genomic loci.^26^, ^27^ As a result, the process of carcinogenesis often leads to clonal and subclonal populations with highly variable DNA methylation patterns.^28^, ^29^, ^30^, ^31^, ^32^ In the context of ovarian cancer, these variable DNA methylation patterns can be found in mature tumours, as well as in precursor lesions of the fallopian tubes.^21^ The PapDREAM microfluidic platform aims to assess the clinical feasibility of using cancer‐specific heterogeneous methylation from routinely collected Pap specimens as an early biomarker for ovarian cancer through ultrasensitive, low‐cost DNA methylation profiling that is amenable to routine screening.

**FIGURE 1:**
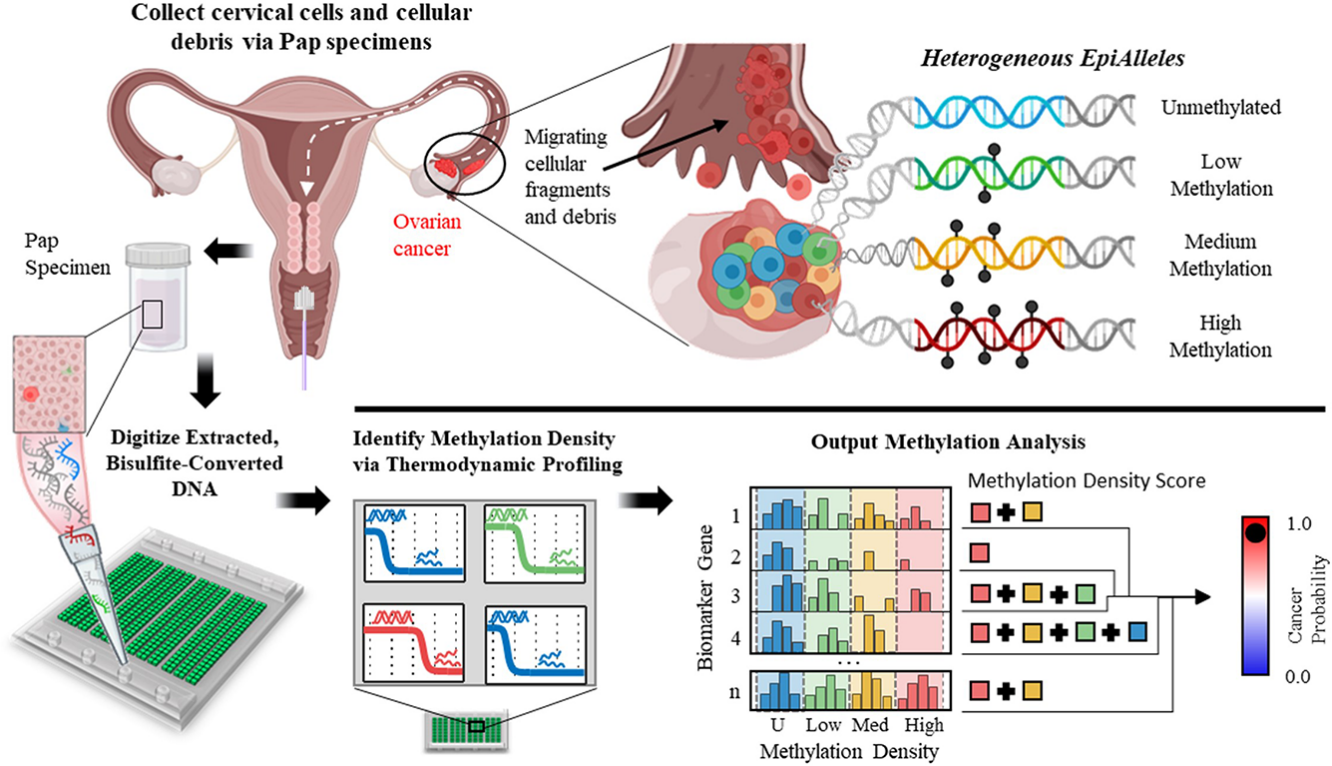
**PapDREAM overview**. Ovarian cancer cells exhibit heterogeneous methylation patterns that could be leveraged as biomarkers for early detection. Cellular fragments and debris from ovarian tumours, which originate in the fallopian tube, migrate to the cervix, where they are accessible via routine Pap specimen collection. The microfluidic HYPER‐melt platform digitizes bisulfite‐converted DNA into thousands of nanochambers to isolate heterogeneous epialleles. Digital high‐resolution melt enables quantitative profiling of methylation patterns on a molecule‐by‐molecule basis. The methylation patterns of a multi‐gene panel are combined into a methylation density score that gives the probability that a given sample contains ovarian cancer DNA.

First, DNA extracted from routinely collected Pap specimens underwent bisulfite treatment to convert unmethylated cytosine residues to uracil, which effectively translates alterations in DNA methylation to changes in primary sequence (Figure 1 and Figure S1). Samples were loaded onto a microfluidic chip comprising four modules, each containing 10,400 1‐nL wells that effectively digitize rare target molecules into individual reaction chambers. Highly parallelized digital PCR (dPCR) was performed on the device using methylation‐preferred and/or agnostic primers to amplify all possible epialleles. The HYPER‐melt platform is able to assess variable methylation patterns by leveraging thermodynamic differences of amplicons derived from bisulfite‐converted template molecules to quantify the fraction of methylated CpGs (or “methylation density”) within a locus on single copies of target DNA. A thermal‐optical platform was developed to perform digital high‐resolution melt (dHRM) analysis to determine the epiallelic identity of each amplicon. Bisulfite‐induced changes in primary sequence from cytosine to uracil alter nucleic acid base stacking^33^ and decrease the thermodynamic stability of double‐stranded DNA (dsDNA), resulting in a measurable shift in the temperature at which a sequence denatures, termed the “melt temperature” T_m_. Melt curves are used to detect methylation patterns for each locus in the biomarker panel. The platform performs this analysis on thousands of individual molecules in parallel in order to produce a quantitative output of molecular variability within a sample. We introduce a comprehensive analysis of the variable methylation patterns across the panel and develop an algorithm to combine the multidimensional information and produce a methylation density probability score that predicts the likelihood that a given sample contains tumour‐derived DNA.

**FIGURE 2:**
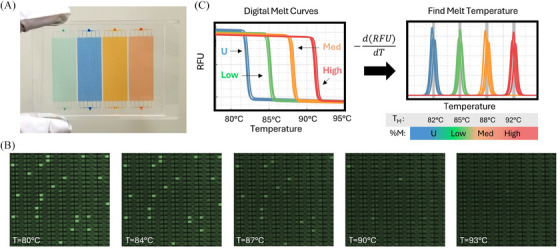
**Thermodynamic profiling of heterogeneous methylation**. (A) The microfluidic device contains four modules of 10,400 1‐nL wells each. (B) After digital polymerase chain reaction (dPCR) amplification, fluorescence images of the entire field of view are captured at increasing temperatures. (C) Digital melt curves are acquired by plotting the fluorescence of individual wells versus temperature. The negative derivative of these curves contains a peak, the melt temperature (T_m_), which corresponds to the methylation pattern of the epiallele. Higher‐density methylation patterns have a higher T_m_ than unmethylated molecules.

Previously, we described the basic principles for the design of the microfluidic device and thermal optical platform for performing highly parallelized dHRM analysis.^25^ Briefly, we developed a custom microfabrication technique for producing a microfluidic array with ultra‐thin polydimethyl siloxane (PDMS) layers that digitize DNA into 1‐nL chambers, while also preventing evaporation during high‐temperature reactions such as PCR. To perform dHRM, we developed a thermal optical platform consisting of a flatbed thermal cycler and wide‐field imager that captures fluorescence images of the entire array at temperature increments of 0.1°C. The current HYPER‐melt platform incorporates several significant improvements to the microfluidic device and thermal‐optical platform to facilitate the interrogation of methylation patterns of thousands of individual molecules with increased efficiency, sample input volume, and throughput. In particular, each microfluidic device comprises four nanoarray modules enabling up to four samples (or DREAMing biomarker assays) to be performed in parallel (Figure 2A). Each module contains 10 000 1‐nL size chambers to digitize DNA. The reaction mix is prepared off‐chip, and then rapidly loaded into the device via vacuum‐based loading. Partitioning oil is then pressurized through the channels to digitize the chambers following our previous technique. Importantly, the cost of materials and reagents for each four‐assay chip is estimated to be less than $25 in total (Table S1).

The principles for discriminating heterogeneous methylation patterns based on melt curve analysis have been laid out previously.^24^ Briefly, a single pair of locus‐specific methylation‐*agnostic* or methylation‐preferred primers enable amplification of all possible methylation patterns (epialleles). Sequence profiling through dHRM is then accomplished by measuring the thermal stability of the methylation‐dependent amplicon sequences by observing the release of a dsDNA saturating dye as a function of temperature. Fluorescence images of the entire chip area are captured simultaneously via a wide field‐of‐view MIL camera during temperature ramping to record the denaturation of each digitized target (Figure 2B). The fluorescence signal is extracted and filtered to produce a melt curve for each amplicon (Figure 2C). The negative derivative of the melt curve with respect to temperature contains a peak, which is termed the “melt temperature” (T_m_) of the sequence, and correlates to the fraction of methylated CpGs (i.e. the “methylation density”) of the amplified target molecules.

Methylation biomarker candidates for early detection of HGSC were derived from our recent discovery work based on genome‐wide methylation analyses of ovarian tumours and ovarian cancer precursor STIC lesions.^21^ To maximize assay specificity in heterogeneous Pap specimens, biomarker loci exhibiting both HGSC‐ and STIC‐specific hypermethylation as well as minimal methylation in healthy gynaecological tissues, including fallopian tube, endometrium and cervical mucosae were specifically selected. From these studies, we identified nine loci that met these criteria and also demonstrated high clinical sensitivity and specificity: *IRX2*, *ZNF154*, *c17orf64*, *c5orf66*, *TBX4*, *miR24*, *TUBB6*, *LRRC32* and *PCDHG* (Figure S2). We designed individual DREAMing assays for each candidate locus according to previously described design criteria.^24^ Each assay was then analytically validated to achieve the specificity criteria described in the Methods (Figure S3), single‐copy sensitivity for methylated control DNA, and single‐ or near‐single‐copy sensitivity for unmethylated control DNA (Figure S4).

To improve confidence in the digital readout and mitigate run‐to‐run variability, we additionally designed and implemented an internal calibration method using control oligonucleotide sequences to quantitatively validate digital amplification efficiency and adjust for thermal variance within and between all modules of the microfluidic device. Unique synthetic control sequences were specifically designed for each respective locus (Figure S5A). The 5′ and 3′ ends of each control sequence were designed to be complementary to each respective primer pair and contain a universal internal sequence complementary to an ROX TaqMan probe. The probe sequence was specifically designed to melt at a temperature lower than the unmethylated DNA for all loci. A fixed number of control sequences were then spiked into each reaction mix before loading into a module of the device to be digitized in discrete wells throughout the module. Digital wells containing sample DNA with the target of interest are amplified and detected via the DNA binding dye (EvaGreen), whereas wells containing the control sequences exhibit fluorescence in both the EvaGreen channel and a separate probe channel to distinguish them from amplified sample templates.

The T_m_ of the control sequences is used to establish accurate and unbiased calibration of the melt temperature to methylation density pattern. For both the unmethylated and fully methylated populations, the differences between the control sequence T_m_ (ΔT_CU_ and ΔT_CM100_) were calculated respectively (Figure S5B). This was achieved for each DREAMing assay using synthetic sequences equivalent to the bisulfite‐converted, fully methylated epialleles and bisulfite‐treated (BST) genomic DNA from healthy women, which was confirmed to be unmethylated at the target loci and used as negative controls. We then established a methylation density discretization function for the conversion between ΔT_m_ and methylation density per locus, to be used for sample data collection (Figure S6). The underlying statistical framework is briefly described in the methods and discussed in our previous work.^24^

### 2.2 Epigenetic profiling of DNA from Pap specimens

We next sought to initially assess the diagnostic potential of the nine biomarker assays by comparing methylation heterogeneity at the corresponding target loci in DNA derived from Pap specimens collected from healthy women during routine gynaecological examinations in comparison to specimens obtained immediately prior to surgery in women with pathologically‐confirmed ovarian cancer. All specimens were obtained from women before gynecologic surgeries following the IRB protocol. The initial cohort included liquid‐based endocervical brush samples from 17 women with advanced stage (FIGO stage III and IV) and 1 woman with early stage (FIGO stage I) ovarian serous carcinoma (median age 56 years) and 25 samples from cancer‐free women (median age 39 years, Table S2), for a total of 9 × 43 = 387 digital assays. DNA was extracted from cell pellets within the Pap specimens and underwent bisulfite conversion prior to analysis on the platform (Figure S1).

**FIGURE 3:**
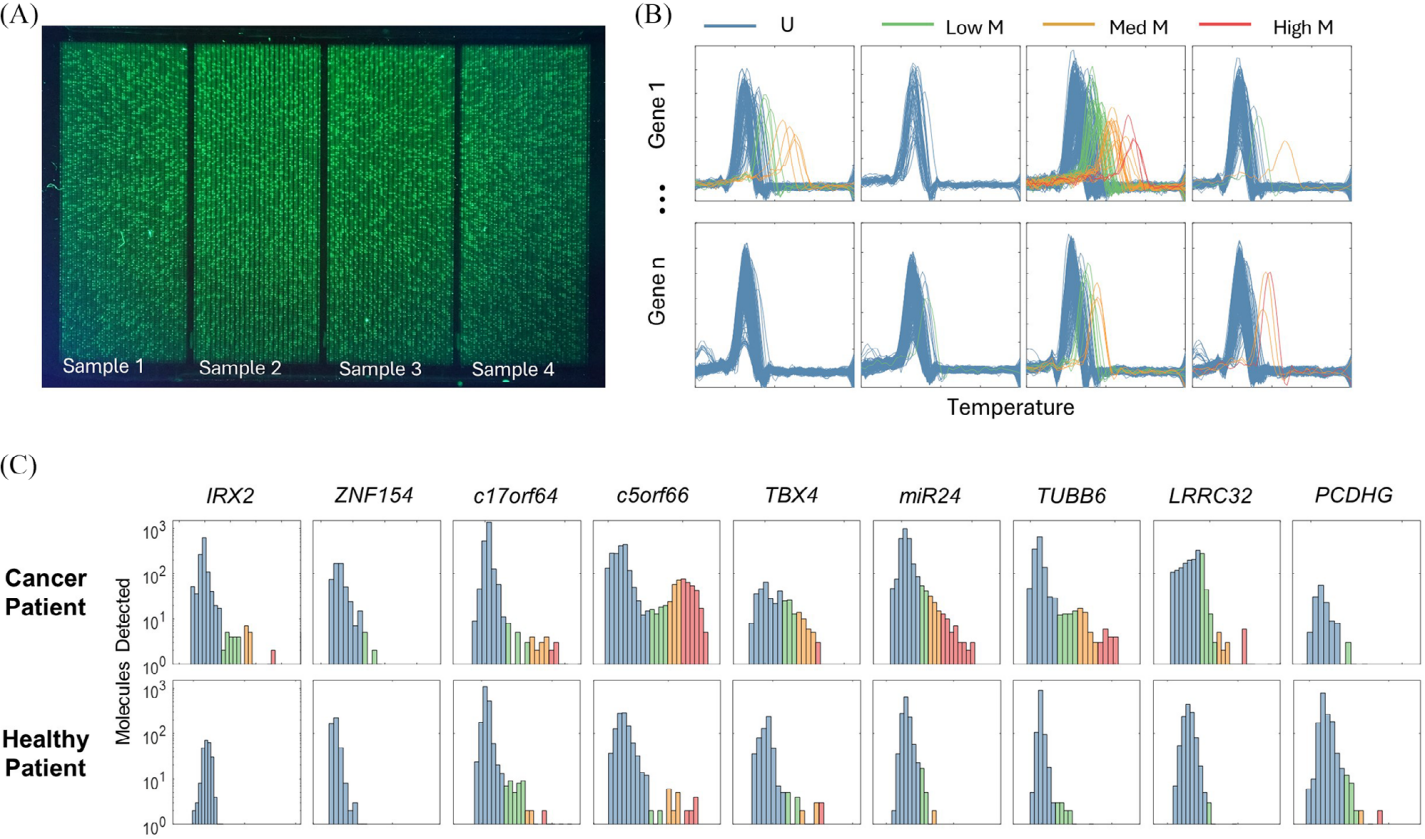
**Nine‐gene biomarker panel on Pap specimens**. (A) One sample per module was loaded onto the device. (B) Melt curves were acquired for each gene in the panel independently. (C) Representative DREAM analyses of each locus in the panel for Pap specimens from cancer and healthy patients, which reports the number of molecules detected at each T_m_, colour‐coded into unmethylated, low, medium and high methylation.

For each locus assay, a reaction mixture containing 5 µL of BST‐DNA from each Pap specimen was loaded into one of the four microfluidic modules on the PapDREAM device (Figure 3A). Following on‐chip PCR, HRM was performed by gradually increasing temperature to denature the target amplicons while monitoring fluorescence. A Matlab script was developed to extract the fluorescence data for each amplicon‐well to produce the resulting melt curves. The negative derivative of the fluorescence with respect to temperature was taken and the location of the peak was defined as the T_m_ (Figure 3B). Methylation levels were then calculated for each amplicon based on the calibration curve for each locus. The detected epialleles for each locus and sample were first stratified into unmethylated molecules, low methylation (∼25%–50% methylation density), medium methylation (50%–75% density), and high methylation (> 75% density) based on the melt temperature of the amplicons. The number of molecules within each methylation stratification was then normalized to the total input DNA of the reaction. Finally, this results in a “DREAM analysis” that provides a histogram of each amplified epiallele present in the sample across the 9‐gene panel. A representative ‘‘DREAM analysis for both a cancer patient and a healthy patient is shown in Figure 3C.

**FIGURE 4:**
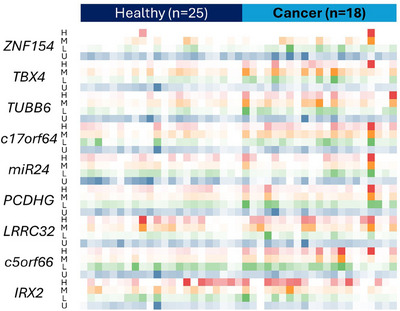
**Pap specimen methylation profiles**. This proof‐of‐concept cohort contains 25 healthy and 18 cancer samples. For each locus, the detected epialleles were binned by methylation density (U, Low, Med and High). A heatmap was generated for each pattern across samples (white = no copies detected).

One key advantage of the HYPER‐melt system used for PapDREAM is the ability to rapidly quantify heterogeneous methylation of individual template molecules. This capability ostensibly provides improved sensitivity for detection, particularly at the early stages of disease when methylation patterns are expected to be more variable and less dense than in later‐stage disease.^34^ Indeed, previous work has shown that incorporation of methylation density analysis can significantly improve the clinical performance of methylation biomarkers,^21^, ^35^, ^36^ but has yet to be extensively utilized due to the challenges of using existing tools to measure intermediate methylation within challenging samples with low target DNA levels. Using our PapDREAM approach, we identified various degrees of heterogeneous methylation in both cases and controls for all of the biomarkers in the panel (Figure 4 and Figure S7). We thus sought to determine if statistical differences in methylation density exist between cases and controls, which might then be exploited to enhance Pap specimen‐based detection of HGSC.

To achieve this, we developed a quantitative single‐molecule methylation density analysis method by first categorizing each detected epiallele according to its methylation density into decile bins (0%–10%, 10%–20%, 20%–30%, … 90%–100%) to build a methylation density histogram for each respective target‐locus. Each bin was then used as a threshold for counting the number of hypermethylated epiallelic molecules present per sample per locus, wherein a 40% methylation threshold compares the number of molecules that exhibited methylation patterns with methylation densities of at least 40%, up to 100%. Furthermore, we sought to investigate the prevalence of molecules containing altered DNA methylation within varying levels of background DNA, nominally derived from healthy tissue. More specifically, liquid biopsy studies commonly report quantification of absolute copy number but vary greatly in methods of standardization or normalization of this value in reference to background DNA populations in plasma. The abundance of heterogeneously methylated loci compared to unaltered DNA has not been well characterized within most liquid biopsy applications, as existing technologies are designed for pattern‐specific detection (e.g. MSP) or low‐mid depth NGS analysis of methylated molecules at the expense of absolute quantification of all methylation patterns/densities within a sample. By contrast, the PapDREAM platform provides simultaneous absolute quantification of methylated and unmethylated epialleles, thereby enabling precise determination of the fraction of epialleles (i.e. the epiallelic fraction) for all possible methylation densities for loci of interest. Since cellular yields from Pap specimens can vary greatly between individuals, we sought to evaluate how these fractional values might affect performance by also calculating the epiallelic fraction of each locus present in each sample.

**FIGURE 5:**
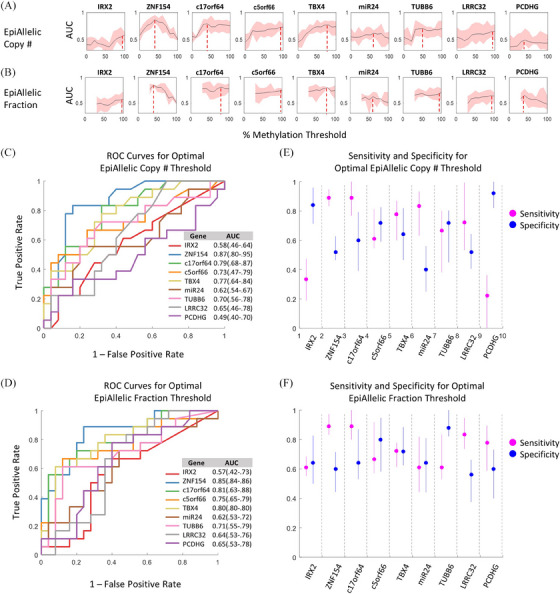
**Performance of individual biomarkers across epiallelic thresholds**. (A) Area under the curve (AUC) optimized for each locus based on different epiallelic threshold levels (with a 95% confidence interval). (B) AUC optimized for each locus based on the number of methylated epialleles normalized to the number of unmethylated molecules. (C) ROC curves for the optimal threshold based on epiallelic copy number for each locus. (D) ROC curves for the optimal threshold based on epiallelic fraction. (E) Sensitivity and specificity with 95% confidence bounds of each locus at the optimal point on the ROC curve in C. (F) Sensitivity and specificity with 95% confidence bounds of each locus at the optimal point on the ROC curve in D.

The epiallelic fraction of low, medium, and high methylated epialleles ranged widely across samples and loci, from as low as 0.00037–0.77 (Figure S8). The area under the receiver operating characteristic curve (AUC) for the training cohort was calculated according to the methylation density threshold applied to each locus. We then compared how each methylation density threshold affects the AUC performance of each respective target locus (Figure 5A). We observed that for 3/9 loci, the fully methylated (> 90% density) epiallelic copy number resulted in the highest AUC. For 3/9 loci and 2/9 loci, a threshold around 40% and 80% methylation, respectively, performed similarly or slightly better than a 100% threshold. Notably, the optimal methylation threshold for *ZNF154* was determined to be 40% with performance significantly declining as the threshold was increased. This finding is qualitatively consistent with a prior study exploring methylation density thresholding for *ZNF154* for blood‐based detection of HGSC.^35^

To explore how normalizing epiallelic fraction to the total number of epialleles in each sample might affect diagnostic performance, we performed similar data analysis to identify optimal epiallelic fraction cutoffs for each locus assay (Figure 5B). The results of this analysis indicated that the effect of density thresholding and optimal AUC values were similar using either the absolute count of hypermethylated epialleles or epiallelic fraction.

We then assessed how the different analysis methods affected the clinical performance of each individual locus. Once the optimal methylation density thresholds were determined, we compared the receiver operator characteristics (ROC) performance of the individual DREAMing assays (Figure 5C,D). Using epiallelic copy number, *ZNF154* performed the best with an AUC of 0.87 (.80–.95), followed by *c17orf64*, *TBX4, c5orf66* and *TUBB6* (0.79 [.68–.87], 0.77 [.64–.84], 0.73 [.47–.79] and 0.70 [.56–.78]). Similarly, *ZNF154* performed the best using the epiallelic fraction method with an AUC of 0.85 (.84–.86), followed by *c17orf64, TBX4*, *c5orf66* and *TUBB6* (0.81 [.63–.88], 0.80 [.80‐.80], 0.75 [.65‐.79] and 0.71 [.55–.79]). We also compared the sensitivity and specificity of each DREAMing assay using the two methods (Figure 5E,F). Though the overall AUC values were similar between the two methods, clinical specificity was notably higher for most loci using the epiallelic fraction values in comparison to absolute copy numbers. Overall, these results indicate that while methylation density thresholding can often improve biomarker performance, each respective threshold value must be determined empirically and will likely be dependent upon the particular biomarker, biospecimen type and intended application.

### 2.3 Methylation biomarker panel performance

**FIGURE 6:**
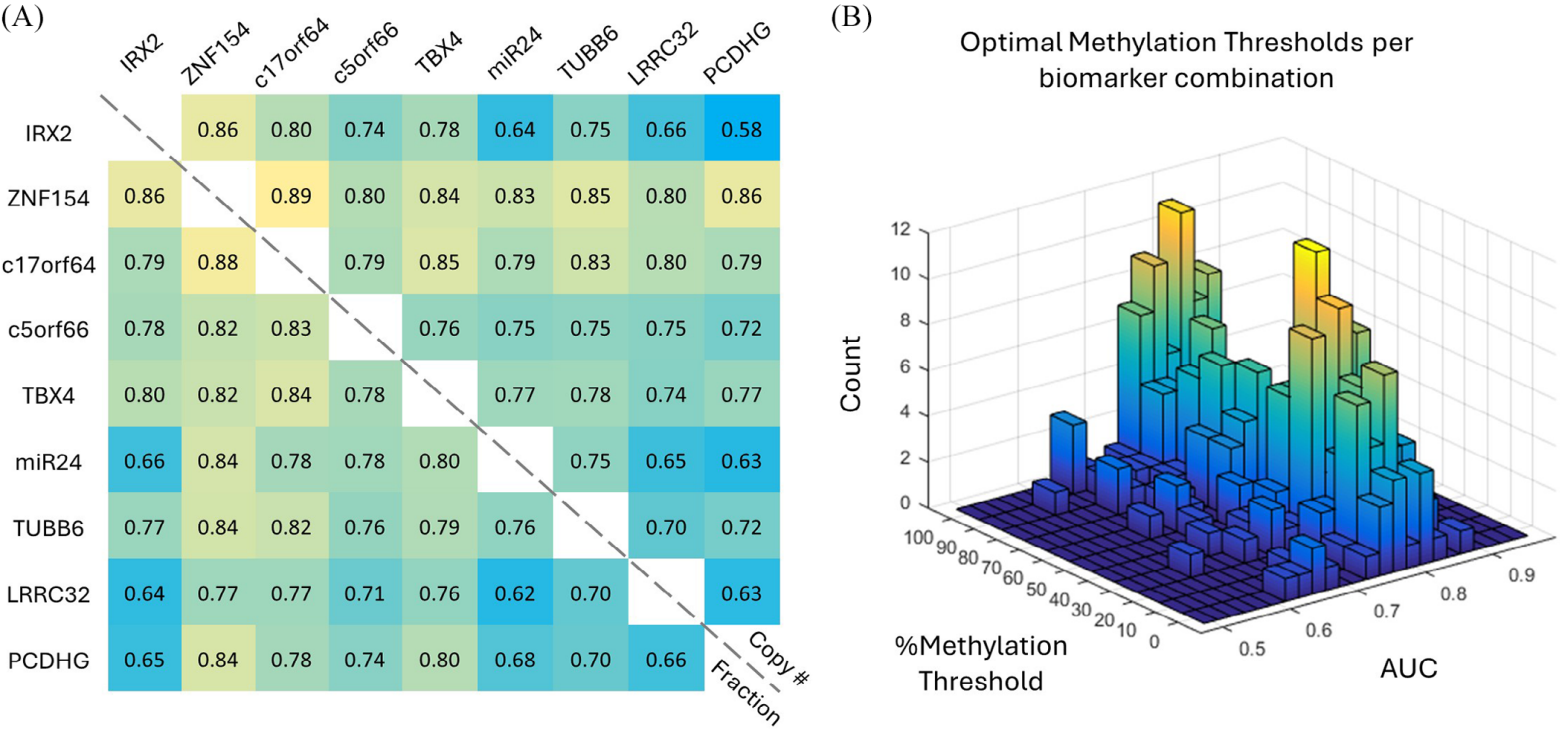
**Effect of intermediate methylation thresholds on the area under the curve (AUC)**. (A) An optimal AUC was calculated by summing any two markers across different epiallelic thresholds. The AUC was calculated based on both the copy numbers per threshold and the epiallelic fraction (normalized to unmethylated molecules). (B) The frequency of methylation threshold levels chosen for each optimal two‐marker combination and the resultant AUC. Higher AUC scores were comprised most frequently of both 40%–60% and > 90% epiallelic thresholds.

Next, we analyzed the performance of the biomarker panel in combination. For each set of loci, we determined the optimal methylation threshold for each marker for each specific combination. Methylation data above each threshold were then combined into a single, unweighted score. ROC and corresponding AUC for each combination were calculated. Figure 6A shows the calculated AUCs for the top combination of any 2 biomarkers in the panel. These results indicated that multigene analysis using only two biomarkers can offer improvements in biomarker accuracy, improving the top AUC to 0.89. We also observed that thresholds based on epiallelic fraction performed similarly to those obtained using epiallelic copy number.

We then further analyzed the significance of various intermediate methylation patterns on the performance of biomarker panels. We compared the methylation density threshold levels that produced the optimal AUC for each single and multigene combination of biomarkers (Figure 6B). Once again, the optimal methylation density thresholds were locus‐dependent and ranged from intermediate methylation (e.g. *ZNF154* 40% and *TUBB6* 50%) to heavy methylation (e.g. *IRX2* > 90% and *c5orf66* > 90%). By comparing the frequency distribution of the methylation thresholds providing the top AUCs per combination, two higher‐frequency clusters of methylation patterns can be observed in the 30%–50% range and 80%–100% range. We believe this result suggests a potential effect of intermediate and heterogeneous methylation in cancer development that warrants further investigation.

**FIGURE 7:**
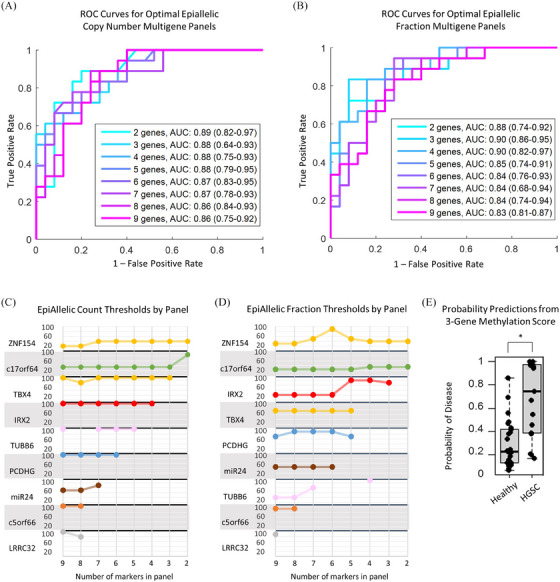
**Clinical performance of biomarker panel**. (A) Receiver operator characteristics (ROC) curves for optimal multigene biomarker panels based on epiallelic copy number and (B) epiallelic fraction with 95% confidence intervals. (C,D) Optimal methylation threshold of each marker per size of a biomarker panel. (E) Probability of disease based on a binomial regression model fit to a three‐gene methylation score. * *p* < .0005 on the Wilcoxon rank sum test.

We next investigated the combination of these markers into a single‐score multigene panel for the detection of all stages of HGSC. Optimal methylation thresholds were determined for each locus in a combination ranging from 2 to 9 genes. Methylation levels above these thresholds were then added together to produce an overall score. ROC curve analysis was used to determine the corresponding AUC for each combination. ROC curves for the optimal combination of 2–9 genes as both epiallelic copy number and epiallelic fraction are shown in Figures 7A,B. Using this simple thresholding method with only four biomarkers, an AUC of 0.90 (0.82–0.97) (specificity 100%, sensitivity 50%) was achieved. With only three biomarkers, an AUC of 0.88 (0.64–0.93) (specificity 100%, sensitivity 55%) was achieved for Pap specimen‐based detection of Pap specimens at all stages of HGSC.

We compared the location of the optimal methylation threshold for each gene across each multi‐marker model (2–9 genes) (Figure 7C,D). Most markers exhibited consistent thresholds across models. Finally, we fit a logistic regression model to the overall three‐gene panel to model the probability of disease for each patient as a function of the epiallelic distribution derived from DNA from their respective Pap specimen. Performance was evaluated using leave‐one‐out cross‐validation (Table S3) to reduce bias, with the entire modelling procedure included in the training step in each iteration of the cross‐validation. The results show that cancer patients are significantly more likely to have methylation density scores that indicate a higher probability of cancer than healthy patients (Figure 7E), suggesting that the PapDREAM method could provide utility for the prediction of ovarian cancer.

## 3 DISCUSSION

To address the unmet need for early diagnosis of ovarian cancer, new tools are necessary to enable the development of molecular assays with sufficient diagnostic performance and suitably low cost for routine early cancer screening applications. In this work, we presented a new, simple and low‐cost method for HGSC detection called PapDREAM that leverages intermolecular epigenetic heterogeneity to identify ovarian cancer from cells obtained from routinely collected Pap specimens. Overall, PapDREAM demonstrated the ability to detect HGSC with 50% sensitivity at 99% specificity.

The PapDREAM approach provides several key advantages for improving Pap specimen‐based ovarian cancer detection. While utilization of Pap specimens minimizes barriers to integration by leveraging an existing clinical workflow, such a strategy necessitates the use of extremely sensitive methods to detect rare cancer‐specific molecular markers that have been shown to be present in these samples at allelic fractions of less than 1.0% and often much lower.^6^ Toward this end, the PapDREAM platform provides single‐molecule sensitivity even among overwhelmingly abundant background DNA from healthy gynecologic tissues. For example, current practice for evaluation of Pap specimens for early detection of cervical cancer requires analysis of a minimum of 15 000 cells.^37^ As DNA from ovarian cancer or precursor tissue is expected to originate from “sloughed‐off cells” and cellular debris that travel down the fallopian tube into the endocervical canal, reliable detection of individual aberrantly methylated epialleles necessitates a method that is able to discriminate these molecules at an analytical specificity of 0.0067% or better. Whereas most other technologies struggle to detect variants below 0.1%, the demonstrated sensitivity of the PapDREAM approach to detect variants as rare as 0.00005%^25^ can readily overcome this challenge. Furthermore, PapDREAM utilizes a simple and intuitive analytical method that can be validated without complex computational analysis tools or proprietary algorithms. Overall, the PapDREAM approach provides a unique, low‐cost and practical workflow that is amenable to routine screening for the early detection of ovarian cancer.

We anticipate that a future clinical workflow using the PapDREAM platform for risk stratification will lead to follow‐up analyses of PapDREAM‐positive specimens to assess additional genetic biomarkers, such as DNA sequence mutations or aneuploidy, or other multi‐analyte approaches, to provide additional robustness in the diagnosis of ovarian cancer. For example, techniques such as PapGene,^6^ PapSEEK^7^ and Duplex sequencing^38^ can be used to evaluate if the presence of DNA mutations can further enhance clinical sensitivity without compromising specificity. PapGene and PapSEEK (with an additional blood draw) are reported to achieve ∼33% and 45% sensitivity at ∼99% specificity, while highly sensitive duplex TP53 sequencing can also achieve ∼44% sensitivity at ∼95% specificity. It is also worth noting that while the current study was focused on leveraging existing clinical workflows (i.e. routine Pap smear collection), the underlying technology of the PapDREAM approach is also compatible with other noninvasively‐collected sample types, such as plasma, which could be analyzed in tandem to improve clinical performance. Indeed, results from the PapSEEK study indicate that paired analysis of Pap and plasma samples significantly improves assay sensitivity for the detection of ovarian cancer. Given the lifetime risk of HGSC is 1.3% in the general population,^39^ implementation of a routine screening test with sufficient positive predictive value is extremely challenging. Therefore, such a technique may be investigated in additional studies targeting women identified as high‐risk who exhibit an HGSC prevalence 10‐ to 40‐fold higher than the general population (e.g. BRCA1/2 germline mutations). Moreover, it is of great interest to determine if PapDREAM can be further extended to detect uterine carcinomas and their precursor lesions.

While these results are promising, several limitations inherent to our current method warrant discussion. First, our results indicate that there exists significant sample‐to‐sample variability between Pap specimens that may arise due to numerous factors. These include variations in sample collection and storage conditions, menstrual status, hormonal use, and patient‐to‐patient variability that affect methylation patterns in the female genital tract. Cellular heterogeneity within pap specimens and its variability in the population, including the prevalence of fallopian tube cells or fragments, has yet to be well‐characterized. Further studies at a clinical trial scale will be needed to isolate these variables and determine their impact on assay performance accordingly. Nonetheless, it is reasonable to assume that incorporating the microfluidic epiallelic profiling approach with a more thorough sampling technique, such as uterine lavage,^40^, ^41^ might provide advantages in terms of improving assay sensitivity even further, as well as potentially mitigating inter‐ and intra‐patient variability. Secondly, the current implementation of the PapDREAM assays requires one module per locus assay, which limits the throughput towards the detection of larger patient cohorts or larger biomarker panels. The method is readily extendible for simultaneous, multiplex biomarker interrogation by carefully tuning assay parameters such as primer design and annealing temperatures for future cohorts. However, interrogation of biomarker panels larger than ∼five would nominally require more complex multiplexing methods, and thus other techniques may be more suitable for preliminary biomarker discovery. Nevertheless, our results support reports by others^42^ that suggest that even a limited number of biomarkers (1–4) are often sufficient to achieve substantial clinical utility, while additional loci may not substantially improve diagnostic accuracy and generally require substantially larger validation studies to prevent overfitting.^43^ Nonetheless, further studies with a larger, preferably longitudinally‐ and prospectively‐collected patient cohort, will be required to fully validate the performance of the PapDREAM panel for the detection of early‐stage ovarian cancer.

## 4 MATERIALS AND METHODS

### 4.1 Device fabrication

A detailed description of the device structure and operation was described previously.^44^ Briefly, the microfluidic chip design comprises four modules per device, each containing 10 040 1‐nL wells. The device consists of a single PDMS pattern layer sandwiched by glass slides and includes PDMS adapters to the inlet and outlet for sample loading. The sample enters the device due to a negative pressure differential created by desiccation for 2 h before loading. The sample is partitioned due to surface tension by pressurizing silicon oil (Sigma Aldrich, 100 cSt) through the channels.

Devices were fabricated using standard photolithography and ultra‐thin soft lithography techniques, as previously described.^25^ Briefly, to fabricate the reusable master mould, a silicon wafer was dehydrated and oxygen plasma treated (Technics PE‐IIA) at 80 watts for 1 min. SU‐8 3050 photoresist (Microchem) was spun on the wafer at 2600 rpm for 1 min. Then the wafer was baked at 95⁰C for 20 min, then exposed to the mask‐aligner at 300 J/cm^2^, developed, and baked at 200°C for 1 hour.

A 15:1 mixture of PDMS (Ellsworth) was spun on the silanized pattern wafer at 500 rpm, while a 6:1 mixture of PDMS was spun on a blank wafer. Both wafers were baked for 6 min at 80°C. The sacrificial layer was then removed, overlaid on the pattern layer, and baked briefly again. A blank glass slide (Ted Pella) was cleaned by rinsing it with water and dried. PDMS (15:1) was spun on the glass slide at 2100 rpm; and then baked at 80°C for 6 min. The two PDMS layers on the pattern wafer were removed jointly. Both the pattern PDMS layer and the blank PDMS on the glass slide were oxygen plasma treated at 40 W for 45 seconds. After bonding, the sacrificial PDMS layer was removed from the pattern. Finally, a tubing adapter and glass coverslip were oxygen plasma bonded to the top surface of the chip. The device was then baked at 80°C overnight, sealed with a piece of thin adhesive tape over the inlet and outlet, and desiccated for a minimum of 2 h before use.

### 4.2 Clinical sample collection and preparation

Endocervical brushed cells were collected in the ThinPrep Pap Test reagent and kept at room temperature until use as previously described (PMID: 23303603, 29563323). DNA was extracted from the collected samples using a Qiagen DNA purification kit from cell pellets. The DNA amount was quantified using a PCR method and subject to bisulfite conversion for the assay. The diagnoses of ovarian cancer were confirmed by corresponding pathology reports (Table S2).

#### 4.2.1 Sample preparation

A simple diagram summarizing the sample preparation procedure is shown below (Figure S1). DNA extraction from Pap specimens was performed with QIAamp DNA FFPE Tissue Kit (QIAGEN) according to the manufacturer's protocol. Briefly, cell from 1 mL of Pap specimen was digested with proteinase K for 1 hour at 55−60°C. DNA was transferred to QIAamp MinElute columns placed in a 2 mL collection tube, washed by washing buffer and ethanol, and consequently eluted into 30 µL of elution buffer. Long interspersed nuclear element 1 (LINE‐1) standards were used to estimate the overall cfDNA copy numbers with 300 nM of forward primer, 5′‐ AGG GTT TTT ATG GTT TTA GGT T −3′, 300 nM of reverse primer, 5′‐ ATC CCT TCC TTA CAC C −3′, spanning 82‐bp regions, and 100 nM of probe, 5′‐ ∖6FAM∖ TTG AAT TGA TTT TGT ATA A ∖MGBNFQ∖ −3′. Cycling conditions were 95°C for 5 min, and 50 cycles (95°C for 30 s, 50°C for 30 s and 72°C for 30 s). PCR was conducted using a PCR buffer containing 16.6 mM (NH4)2SO4, 67 mM Tris (pH 8.8), 10 mM β‐mercaptoethanol and magnesium chloride to yield a final magnesium concentration of 6.7 mM, 200 µM of each deoxynucleotide triphosphate (dNTP, MilliporeSigma) and 1 U of Platinum Taq polymerase (Thermo Fisher Scientific). The resulting DNA was bisulfite converted using the EZ DNA Methylation‐LightningTM Kit (ZYMO RESEARCH) according to the manufacturer's instructions and eluted into a final volume of 30 µL. DNA yields were quantified by LINE‐1 quantification assay as described above. All synthetic control DNA was purchased from Integrated DNA Technologies (IDT) and used at concentrations suggested by the manufacturer.

### 4.3 DREAMing assay validation

Methylation‐preferred primer pairs were designed for nine candidate loci based on previously described criteria^24^ (Table S4). In brief, design constraints were as follows: (I) inclusion of 0, 1 or 2 CpG dinucleotide in each primer toward the 5′ end^45^; (II) primer melting temperature near 60°C and within 2°C of each other; (III) no significant hairpin or primer dimer formation. All primers were ordered from IDT. Primer pair candidates were evaluated experimentally through the analysis of cycle thresholds (Ct) values and melt curve profiles based on two major criteria: (1) Ct values of the primer dimer are equal to or later than 40; (2) the ability to produce clearly distinguishable single‐peak melting temperature differences in unmethylated versus methylated controls.

Assay validation on bulk was conducted with synthetic methylated DNA and bisulfite‐converted control genomic DNA. First, adherence to specificity criteria in non‐template control reactions was assessed (Figure S3). Next, approximately one genomic copy equivalents of methylated DNA and 100 or 500 copies of unmethylated control genomic DNA were mixed into a well of a 96‐well microtiter plate. The presence of clearly distinguishable two melting peaks indicates the successful amplification of the methylated DNA among 100 or 500 copies of genomic background (Figure S4).

### 4.4 Digital PCR and digital melt

Devices were placed on a flatbed heater (Biorad Proflex) with FC‐40 oil between the glass and the heater surface. PCR protocol was 95°C for 5 min, followed by 60 cycles of (95°C for 30 s, Ta for 30 s, and 72°C for 30 s), where Ta varied based on the target locus. Following PCR, devices were taken to the digital melt setup and secured on the heater via adhesive tape (3 M). The heater ramped at 0.05°C/s and images were taken with a full‐frame camera (Sony MILC) every second with 0.8 s exposure. A 480 nm LED array (Thorlabs) was used to illuminate the device, and fluorescence emission passed through a 570 nm bandpass filter (Edmunds Optics). A detailed description and schematic of the thermal‐optical platform were previously described.^25^ The thermal uniformity across each module was estimated from the melt temperatures of the synthetic control sequence amplicons. The average standard deviation of melt temperature of these amplicons ranges from 0.30 to 0.36°C from low to high‐temperature range (Figure S9). Devices were imaged on a typhoon scanner (GE, 590 nm laser, 630 nm bandpass filter) to image the fluorescence of the control sequence probes.

### 4.5 Image processing and data analysis

Melt images were aligned after collection to correct for any thermally induced movement via an open‐source automated program, Automated Image Registration.^46^ Fluorescence information was extracted from the images using Matlab as previously described.^25^

Fluorescence intensity values within each were averaged within 0.3°C temperature intervals. Melt curves were generated by performing a low‐pass and Savitzky‐Golay filter on each well. The melt temperature of each amplicon was found by taking the negative derivative of the signal and identifying the location of the corresponding peak(s). Positive peaks were determined by setting an arbitrary peak height threshold of approximately the average + 11 times the standard deviation of the baseline ΔRFU.

Reference probe images were analyzed via the same mask generation, and fluorescence values within each well were averaged to calculate the signal. Positive wells for the reference molecule were identified via an arbitrary brightness threshold. The average melt temperature of the reference wells was used to set objective melt temperature thresholds which correlated the methylation density of the sample molecules. ROC curves were calculated using built‐in functions in Matlab.

### 4.6 Epiallelic variant calling statistical probability

To estimate the precision of melt temperature‐based epiallelic variant calling, we obtained synthetic sequences representative of unmethylated, 37.5% methylated, 64.5% methylated, and 100% methylated epialleles of a representative locus, *c17orf64*. Each epiallele was digitized and amplified on the microfluidic device, and the T_m_ was collected (Figure S10). We then derived an in silico melt temperature model using the Unified thermodynamic library through uMelt.^47^ After verifying that model‐derived melt temperatures were well correlated with experimental T_m_’s, we used the in silico model to estimate variation in melt temperature due to different permutations of each methylation density.^24^ An overall standard error was calculated as the geometric mean of 1) the per‐epiallele standard deviation of the experimental T_m_ data, 2) the theoretical deviance of the same epialleles, from the uMelt model, and 3) the standard deviation calculated over permutations of the original epialleles. Finally, assuming that melt temperature follows a normal distribution with the overall standard error from above, we computed melt temperature likelihood functions for methylation density.

To estimate the influence of age on the presence of medium and high methylation, we fit a linear regression model comparing the epiallelic fraction detected and patient age for each marker. Coefficients of determination of < 0.25 for each marker suggest that age does not strongly influence the methylation of the markers in this study (Figure S11).

## AUTHOR CONTRIBUTIONS

Tza‐Huei Wang, Ie‐Ming Shih, Tian‐Li Wang and Thomas R. Pisanic designed the study and secured funding. Chih‐Ming Ho, Amanda N. Fader, Rebecca Stone, James S. Ferris, Anna Beavis, Kimberly Levinson and Stephanie Wethington collected the patient samples. Yang Zhao prepared the samples and conducted experiments relating to the sample preparation protocol. Thomas R. Pisanic, Yang Zhao and Christine M. O'Keefe designed the primers and control sequences. Christine M. O'Keefe designed and conducted the microfluidic experiments, analyzed the data and prepared the original draft of the manuscript. Yang Zhao, Thomas R. Pisanic, Tza‐Huei Wang, Leslie M. Cope and Ie‐Ming Shih reviewed and edited the manuscript.

## CONFLICT OF INTEREST STATEMENT

The authors declare no conflict of interest.

## ETHICS STATEMENT

The JHU IRB board approved the study which was for the use of tissue and biospecimens acquired from consented patients.

## Supporting information

Supporting Information

## Data Availability

All data associated with this study are in the work or the Supporting Information.
