# Supplementary material for: Single‐molecule epiallelic profiling of DNA derived from routinely collected Pap specimens for noninvasive detection of ovarian cancer
Source: Clin Transl Med. 2024 Jul 31;14(8):e1778. doi: 10.1002/ctm2.1778 (PMC11290349; doi:10.1002/ctm2.1778)
Supplement: Supplementary file 1 — Supporting Information [file CTM2-14-e1778-s001.docx]

Supplementary Information for Intermolecular epiallelic profiling of DNA derived from routinely collected Pap specimens for noninvasive detection of ovarian cancer

**Authors:** Christine M. O’Keefe^1^, Yang Zhao^1^, Leslie M. Cope^2,3^, Chih-Ming Ho^4,5^, Amanda N. Fader^6^, Rebecca Stone^6^, James S. Ferris^6^, Anna Beavis^6^, Kimberly Levinson^6,7^, Stephanie Wethington^6^, Tian-Li Wang^2,6,8^, Thomas R. Pisanic II^9*^, Ie-Ming Shih^2,6,8*^, Tza-Huei Wang^1,2,9,10*^


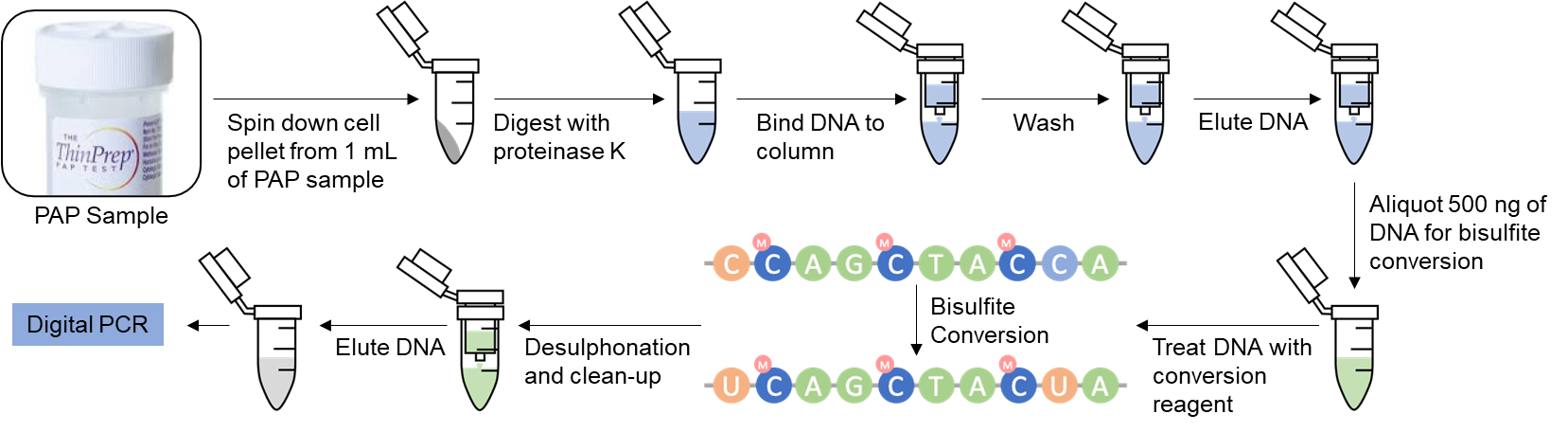


**Supplementary Fig. S1. Sample Preparation Procedure**

A spin-column based method was used to extract DNA from Pap specimens. Extracted DNA then underwent bisulfite treatment to have unmethylated cytosine (C) converted to uracil (U) and further thymine (T) while leaving the methylated cytosine intact. DNA is now ready for the downstream applications.


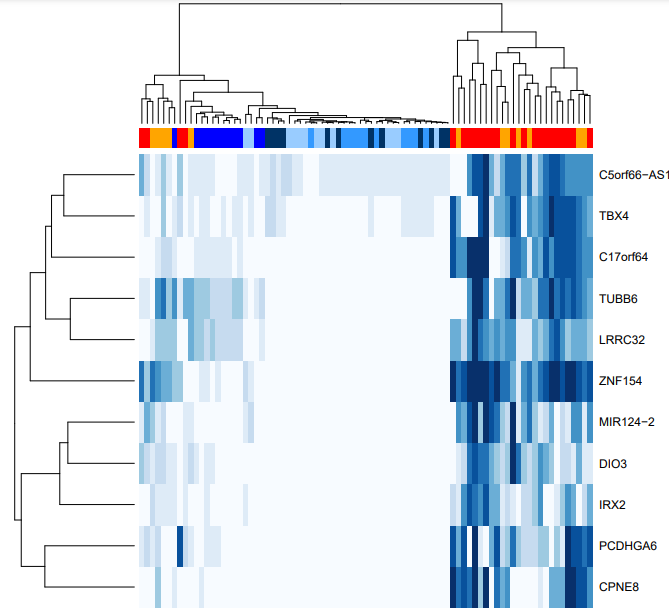


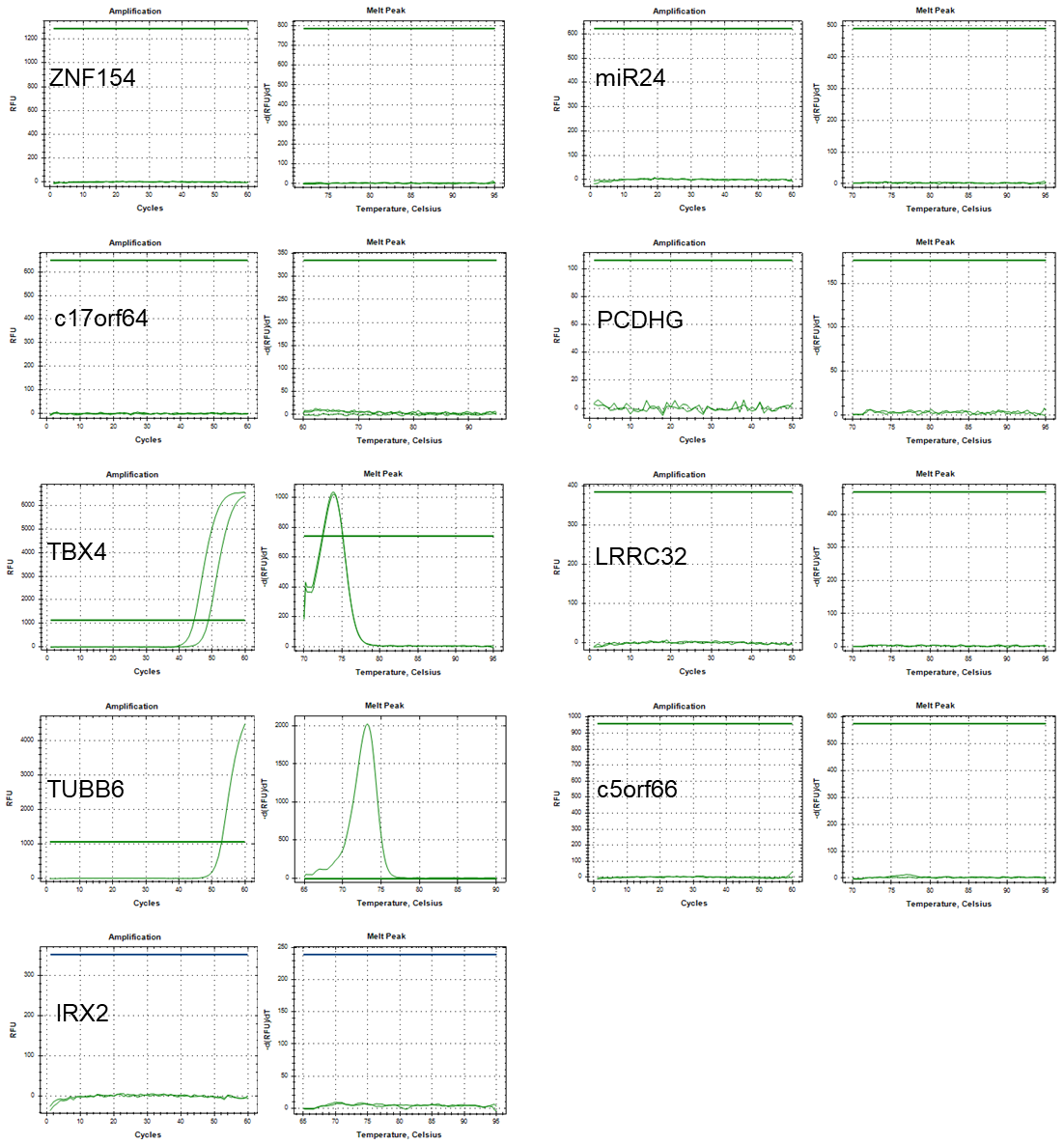


**Supplementary Fig. S2. Candidate Biomarkers Methylation in Tissue.**

Methylation levels of candidate biomarkers assessed on the Infinium 850K array on HGSC and STIC tissue as well as healthy gynecological tissue, including fallopian tube, endometrium and cervical mucosae. Candidate loci demonstrated low (β < 0.2) methylation levels in healthy tissue and high discrimination between tumor and adjacent normal tissue.

HGSC

EM

FM

CM

STIC – Adj. FTE

STIC

**Supplementary Figure S3.**

No template control analysis in bulk. Each primer set was assessed to ensure no amplification before cycle 40. If any amplification occurs later, the T_m_ must be below 75 °C.


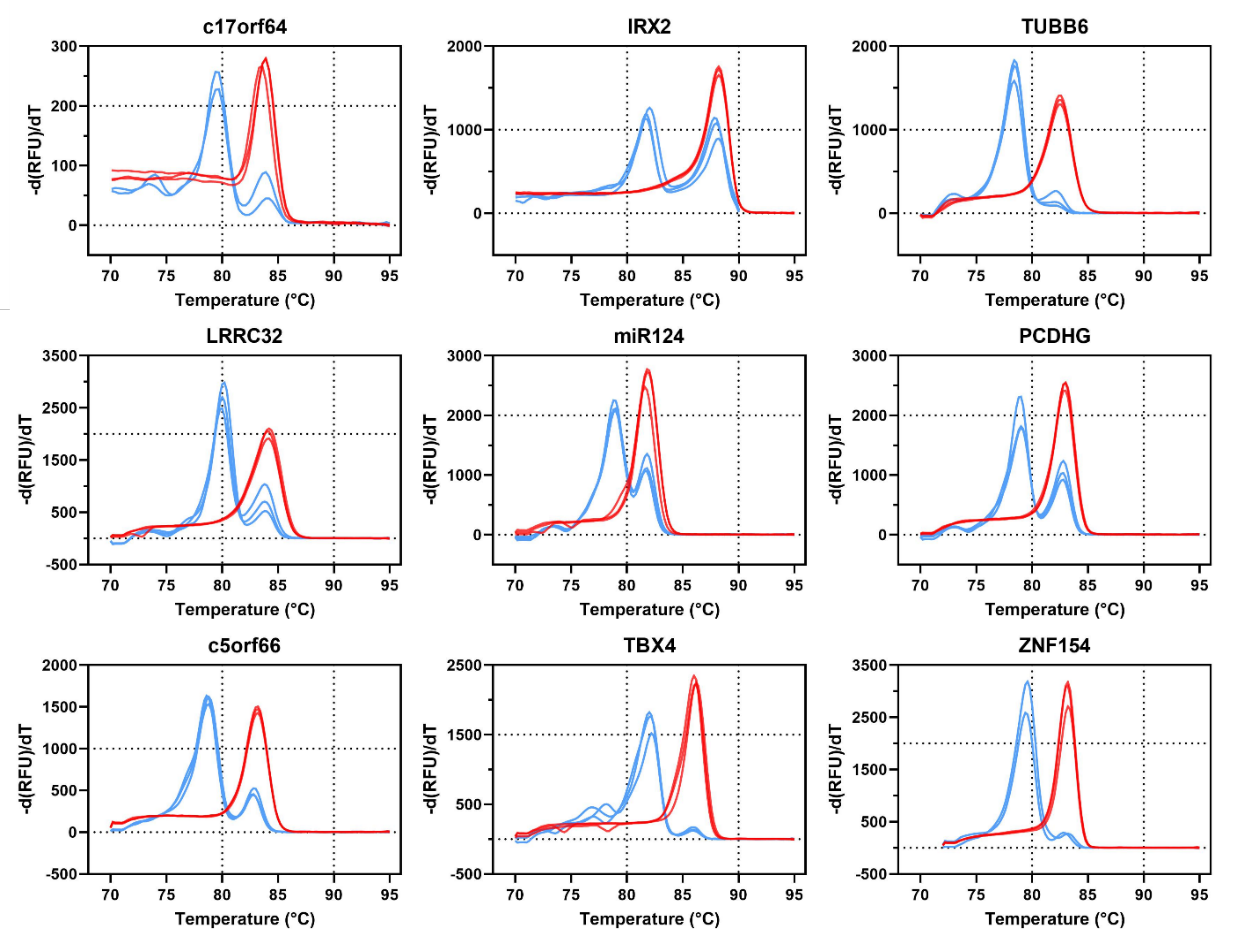


**Supplementary Fig. S4. Assay Validation**

Bulk validation. About one methylated synthetic DNA is added to a single well (in red) or mixed with a certain copy of unmethylated genomic control DNA (in blue). The number of unmethylated genomic background DNA: *c17orf64*: 5 copies. *IRX2*, 50 copies. *TUBB6*, *LRRC32*, *miR124*, *PCDHG*, 100 copies. *c5orf66*, *TBX4*, *ZNF154*, 500 copies.


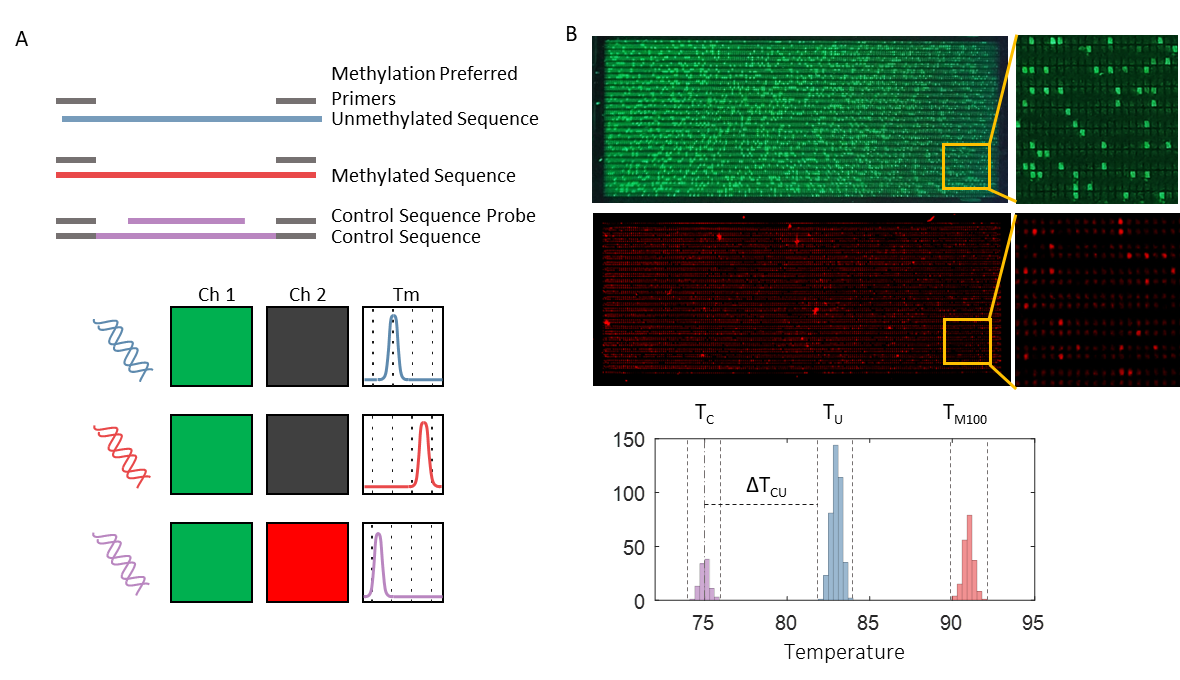


**Supplementary Figure S5.**

(A) Methylation preferred primers are design to amplify all possible methylation patterns of a locus. A control sequence is designed to be complementary to the primers and to a ROX TaqMan probe. Following amplification, all molecules will exhibit intercalating-dye fluorescence signal in the intercalating-dye channel (Ch 1). Nanochambers containing the control sequence can be identified via the ROX probe channel (Ch2). (B) Methylation stratification calibration is performed using the T_m_ of the probe sequences as a reference. Control unmethylated DNA and synthetic sequences representative of full methylated bisulfite-converted DNA are used to establish T_m_ edges for discretization into epiallelic bins. Intermediate edges are interpolated assuming a linear relationship based on thermodynamic models.


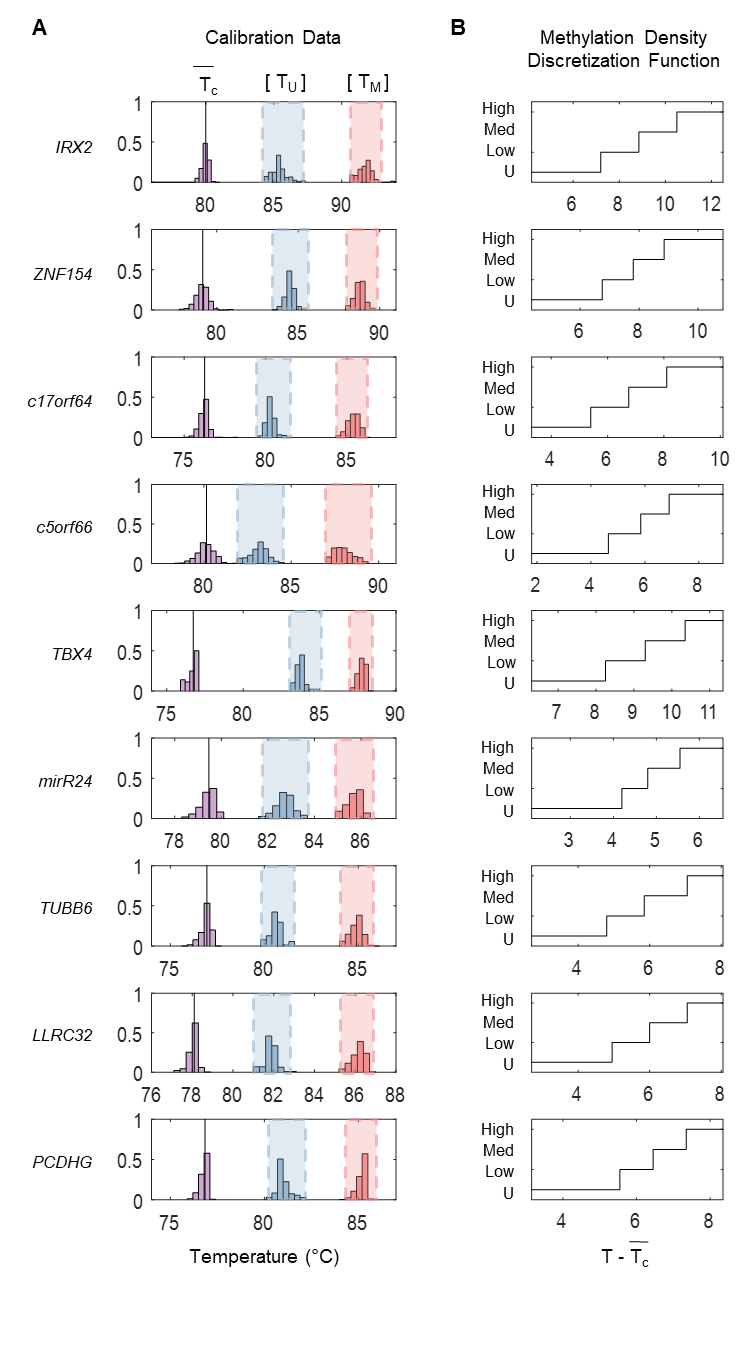


**Supplementary Fig. S6. Methylation Calibration**

(A) Calibration experiments for each assay on the microfluidic device using control sequences, genomic unmethylated healthy control, and synthetic sequences representative of fully methylated epialleles. (B) Methylation density discretization function applied to clinical samples to stratify epialleles into U, Low, Medium, and High bins.


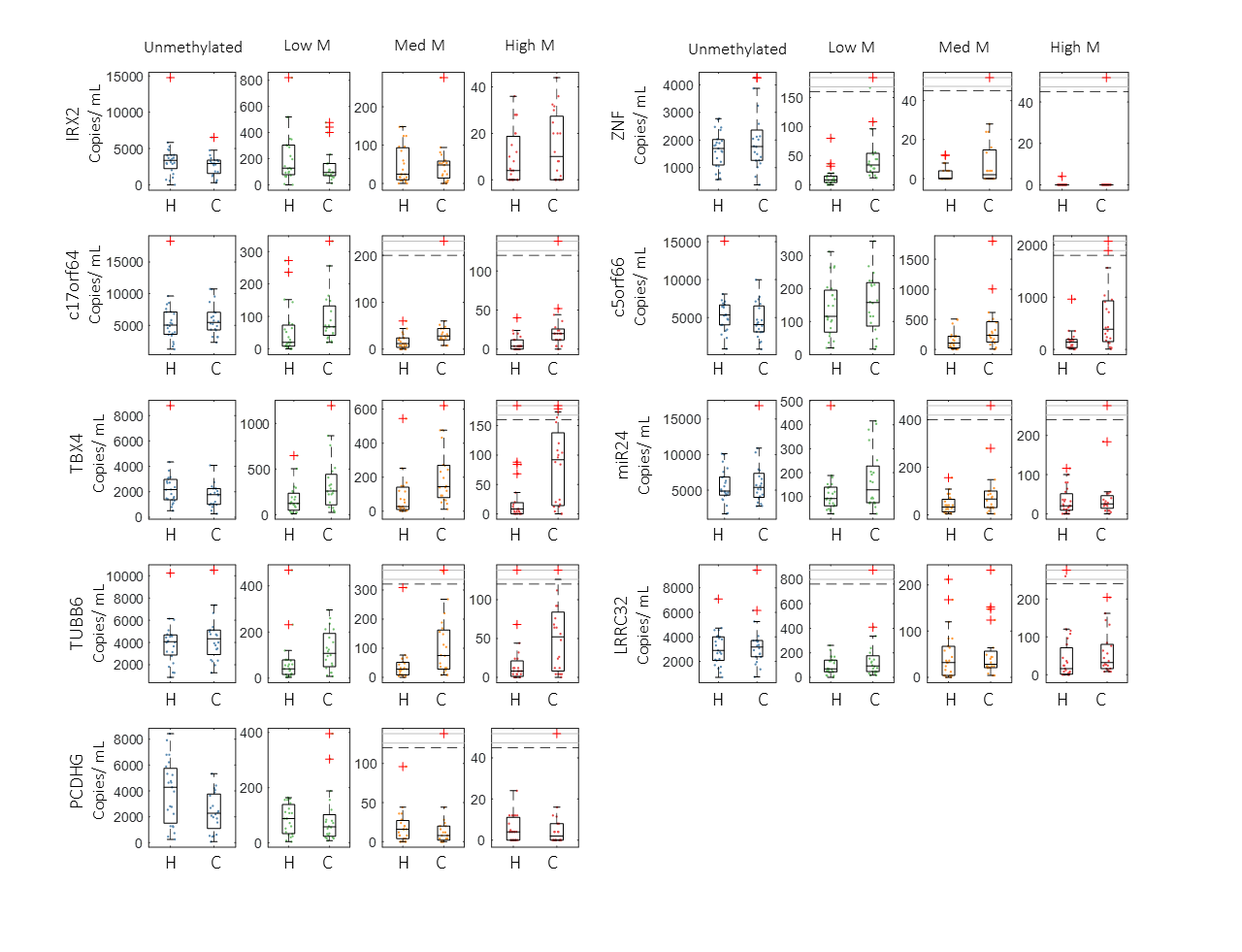


**Supplementary Fig. S7. Methylation Levels per Biomarker**

The number of epialleles for each locus stratified into Unmethylated, Low, Med, High bins. Box and whisker plots summarize trends for healthy (H) and cancer (C). Dashed lines represent axis breaks. ‘+’ represent outliers.


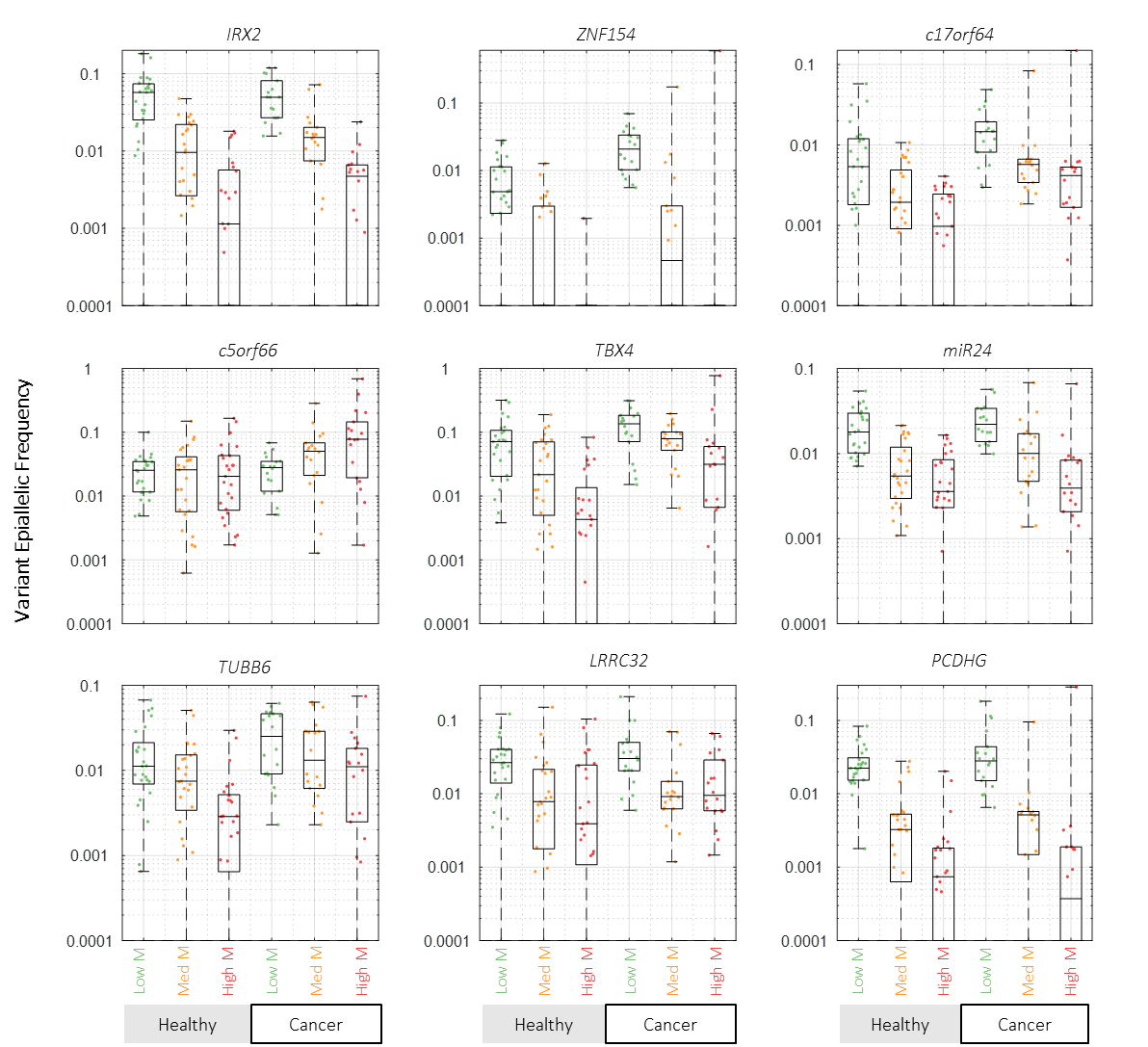

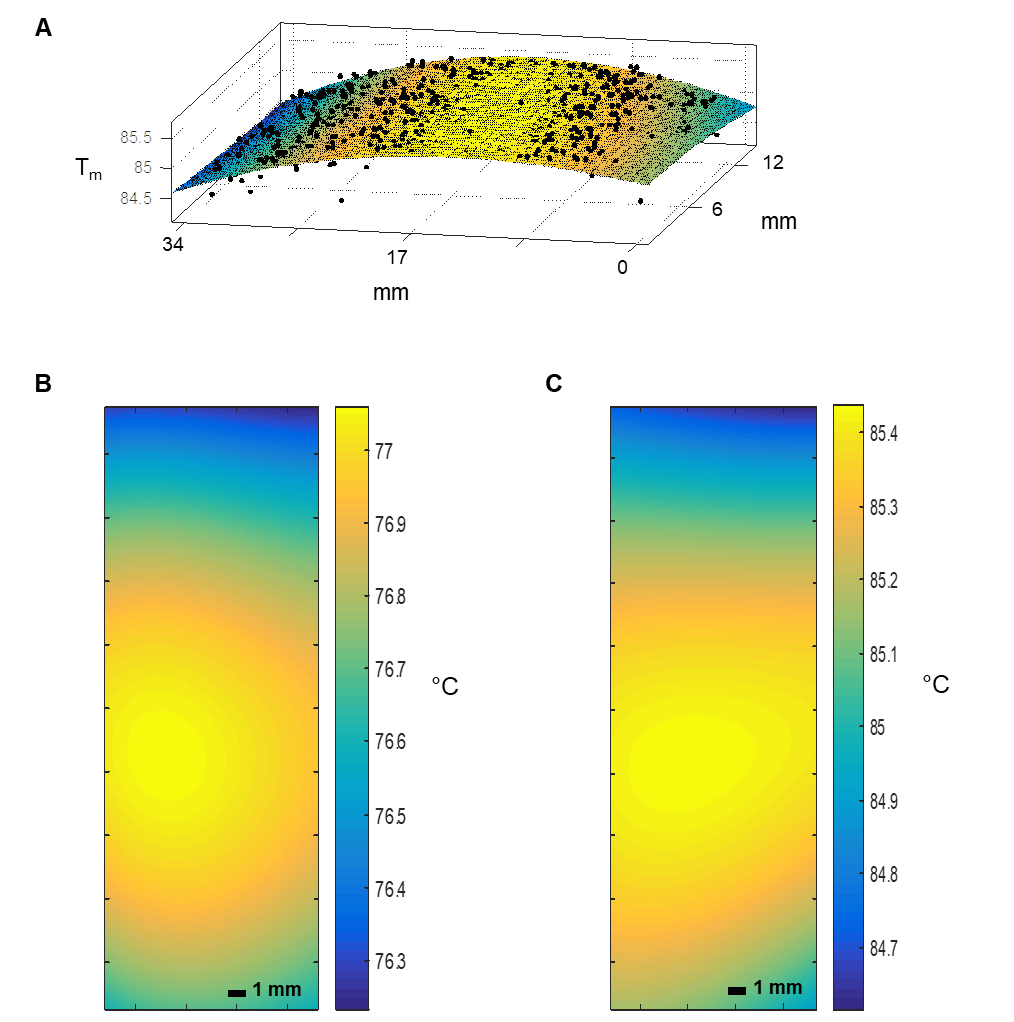


**Supplementary Figure S8.**

The Variant Epiallelic Frequency detected for each biomarker was calculated by normalizing the copy number of methylated epialleles (low, medium, or high) to total epialleles detected for each sample. The range of VEFs detected (above 0) was 0.000371 to 0.7708.

**Supplementary Figure S9.**

(A) Thermal surface model fit to melt temperature locations of calibrator sequence amplicons. (B) Thermal uniformity of single module at low temperature range. Surface was fit to T_m_ distribution of calibrator sequence amplicons. (C) Thermal uniformity of single module at high temperature range. Surface was fit to T_m_ distribution of calibration synthetic methylated amplicons. The average thermal standard deviation across an individual module is 0.30 °C at the low temperature range and 0.36 °C at the high temperature range.


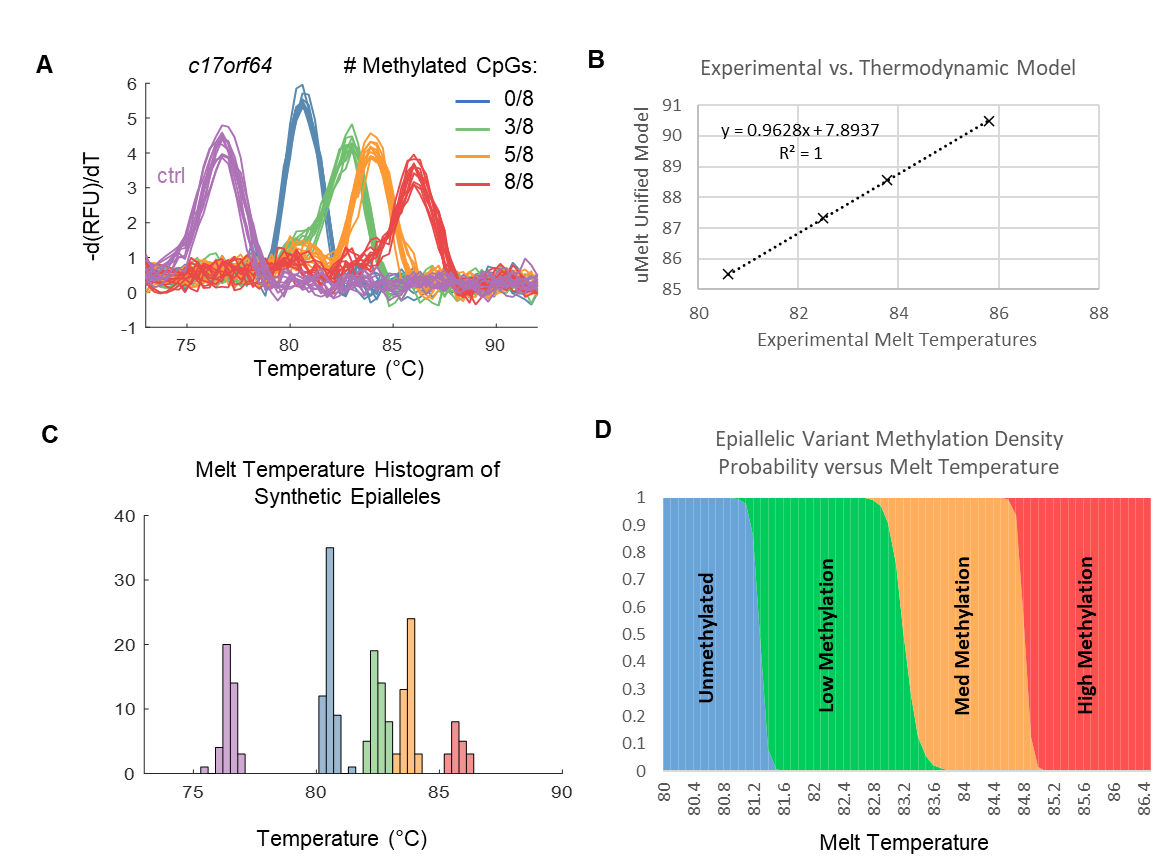


**Supplementary Figure S10.**

(A) Melt curves from synthetic sequences representative of epialleles of *c17orf64* on the microfluidic device. (B) Linear estimate of relationship between experimental melt temperatures and predicted temperatures from the Unified thermodynamic library. (C) T_m_ histograms of the calibration data on the microfluidic device. (D) Probability of epiallelic variant call based on melt temperature.

**Supplementary Fig. S7. Statistical Model of Epiallelic Variant Probability**

(A) Digital melt curves from synthetic sequences representative of epialleles of *c17orf64* on the microfluidic device. (B) Linear estimate of relationship between experimental melt temperatures and predicted temperatures from the Unified thermodynamic library. (C) T_m_ histograms of the calibration data on the microfluidic device. (D) Probability of epiallelic variant call based on melt temperature.


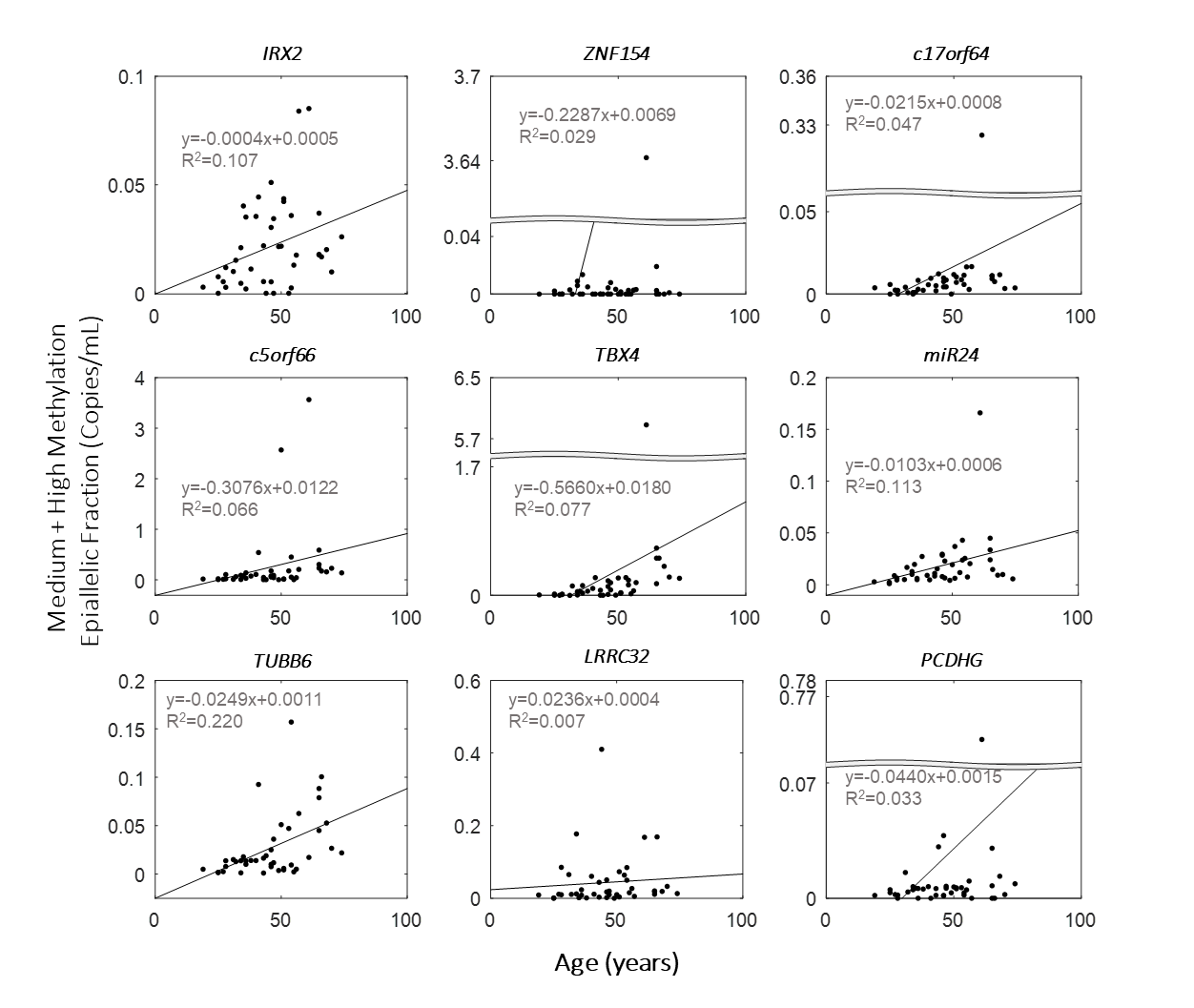


**Supplementary Figure S11.**

Epiallelic fraction of combined medium and high methylated molecules for each biomarker compared to the age of the patient. We fit a linear regression model to assess the relationship between age and methylation for each marker. The low coefficient of determination suggests that age does not have a strong effect on the presence of medium or high methylated epialleles.


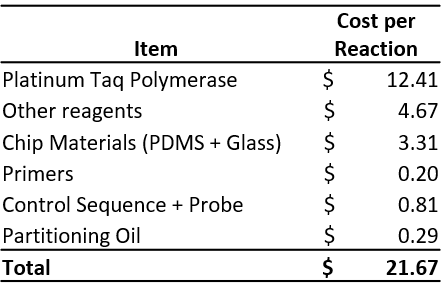


**Supplementary Table S1. Estimated Cost Per Microfluidic Reaction**


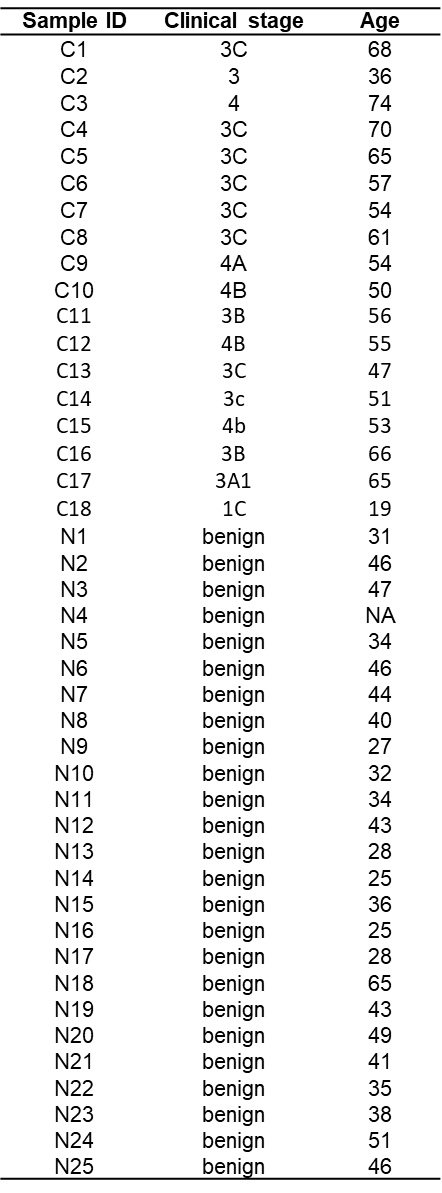


**Supplementary Table S2. Clinical Characteristics of Pap Specimens**


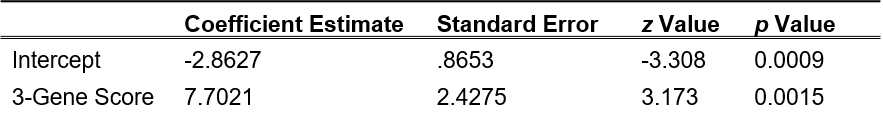


**Supplementary Table S3. Logistic Regression Model on All Samples**


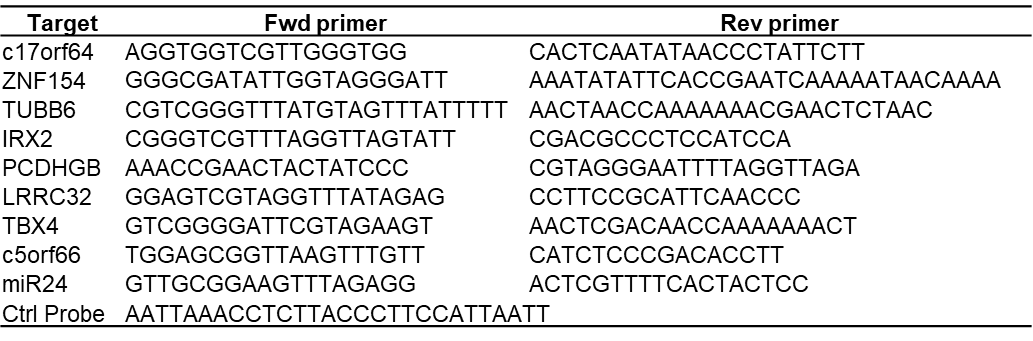


**Supplementary Table S4. Primer and control probe sequences**
